# Late-Onset Hypogonadism as Primary Testicular Failure

**DOI:** 10.3389/fendo.2019.00372

**Published:** 2019-06-12

**Authors:** Du Soon Swee, Earn H. Gan

**Affiliations:** ^1^Endocrine Unit, Royal Victoria Infirmary, Newcastle upon Tyne, United Kingdom; ^2^Department of Endocrinology, Singapore General Hospital, Singapore, Singapore; ^3^Institute of Genetic Medicine, International Centre for Life, Newcastle University, Newcastle upon Tyne, United Kingdom; ^4^South Tyneside and Sunderland NHS Foundation Trust, Newcastle upon Tyne, United Kingdom

**Keywords:** late-onset-hypogonadism, hypogonadism, andropause, obesity, testosterone, aging

## Introduction

Testosterone (T) therapy has garnered widespread public enthusiasm and media attention due to its potential role in age-related T decline in men, commonly known as late-onset hypogonadism (LOH), andropause, or low T syndrome. The serum T concentration gradually declines across the lifespan and the symptoms between aging and hypogonadism overlap. These have led to the speculation that a causal relationship might exists between age-related reduction in serum T concentration and symptoms commonly seen in aging. However, it remains uncertain if T therapy could ameliorate symptoms associated with LOH, without significant risks. Despite the lack of clinical evidence and long term safety data, prescribing rates of T therapy have skyrocketed in many countries (1, 2), leading to efforts by regulatory authorities to limit such inappropriate prescribing practice (3).

Importantly, the fundamental question of what constitutes clinically significant LOH was largely unaddressed until recently. Heterogeneity in definitions of LOH and the use of specificity-limited immunoassays for T measurements in many previous epidemiological and interventional studies have precluded robust comparisons across studies (4). Due to the expanding aging population, LOH is becoming an increasingly important topic. We reviewed the evidence from recent population-based studies and intervention trials to provide better understanding of the diagnosis, pathophysiology, and management for LOH.

## Pathophysiology of T Decline in AGING

The testicular function undergoes natural decline with age. Compared to younger men, healthy older men has 40% less Leydig cell mass and a corresponding rise in luteinizing hormone (LH) concentration (5). Decreased testicular T production was also observed in aged Leydig cells, following diminished LH-stimulated cAMP production, and reduced downstream steroidogenic enzymatic activity (6). On the other hand, aging is associated with changes in LH secretory pattern. A reduced T production and frequent, small irregular LH pulses was observed in healthy older men (7), despite preservation of pituitary gonadotrophs' response to exogenous gonadotropin-releasing hormone (GnRH) (8).This suggests age or factors associated with aging reduced negative feedback inhibition by T. An ensemble-based analysis also predicted a >30% fall in GnRH output in healthy older men (9). However, a recent study has demonstrated that healthy older men without late-onset hypo-gonadism (LOH) have preserved hypothalamic response to kisspeptin-54 and pituitary response to GnRH, with impaired testicular response as compared to younger men (10). This suggests that primary testicular failure accounts principally for the normal aging-related decline in T production. In majority of healthy older men, the compensatory increase in gonadotrophins serves to maintain T levels within eugonadal ranges (11).

The pathophysiology of LOH is complicated by comorbidities associated with aging. The development of chronic illnesses, including diabetes, cardiovascular disease and inflammatory disorders, is associated with a contemporaneous accelerated rate of aging-related T decline, ranging between 1.5- and 3.6-fold compared to men who remain disease-free (12, 13). Furthermore, excess adiposity exerts potent suppressive effects on the HPT axis. Individuals with BMI ≥30 kg/m^2^ are at 13-fold increased risk of LOH compared to those with BMI <25 kg/m^2^ (14). Overall, men with comorbidity and/or obesity failed to exhibit compensatory rise in LH levels which would otherwise expected in healthy non-obese men suggesting a significant disruption at the hypothalamic-pituitary level which compromises T production (15).

**Figure 1:**
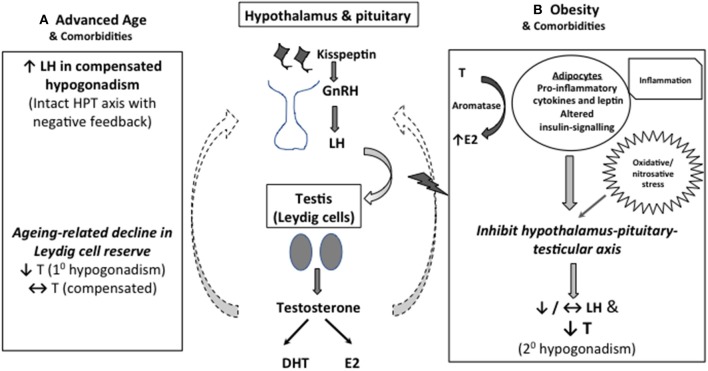
Mechanistic explanation for low serum T in middle-aged and older men. **(A)** As Leydig cell reserve decline with aging, compensatory rise in luteinising hormone (LH) occurs to maintain circulating testosterone (T) concentrations (compensated hypogonadism). In more advanced state, elevated LH can no longer overcome the diminished testicular function, leading to overtly low T levels (primary hypogonadism). **(B)** Obesity is the predominant cause of functional suppression of hypothalamic-pituitary-testicular (HPT) axis in middle-aged and older men, manifesting as failure of LH response to low T (secondary hypogonadism). Multimorbidity is also associated with both primary and secondary hypogonadism, albeit to a lesser degree. Excess adiposity has been linked to altered insulin signaling, oxidative stress and increased pro-inflammatory cytokines and leptin levels, which act in concert to suppress the central HPT axis. Adipose tissues also express aromatase which convert testosterone to estradiol, especially in the inflammed state, exerting inhibitory effects on the HPT axis.

Consonant with that, obesity has been shown to be the most common factor associated with the development of low T in middle-aged and older men (11). The pathogenic role of excess adiposity has been postulated to be linked to several adipose tissue-derived factors, including pro-inflammatory cytokines and leptin, and altered insulin-signaling, which act in concert to produce central inhibitory effects on the HPT axis, leading to secondary hypogonadism (16–18). Interesting, obesity also increase oxidative/nitrosative stress leading to nitroso-redox imbalance and male sexual dysfunction (19). The potential mechanisms underpin the development of LOH is depicted in Figure 1.

## AGING-related Decline in T Concentrations

Serum total T concentrations were historically thought to decline at a rate of 1–2% per annum from 4 to 5th decade onwards (20, 21). One of the population-based studies demonstrated that >50% of men aged ≥80 years had T level in hypogonadal range, as defined by <2.5th percentile for young men (<11.3 nmol/l) (22).

However, accumulating evidence from newer studies suggest that age-related fall in serum total T is closer to 0.5% per year (12, 15, 23), and healthy older men actually experience minimal changes in T levels. A community-based longitudinal study from South Australia showed that the rate of decline of total T concentrations in a subset of men without chronic illnesses was a non-significant 0.27% per year (12). In another study, no appreciable change in serum T up to 8th decade of life was observed among men with self-reported very good to excellent health (24). In European Male Aging Study (EMAS), 2,736 men aged ≥40 years were followed up for an average of 4.4 years, and >80% of men in their 7–8th decade continued to have normal T values (11). Therefore, LOH is less prevalent than previously thought, and low T in older men is mostly related to co-existing medical conditions and obesity.

## The Challenges in Diagnosing LOH

LOH has conventionally been defined as low serum T in older men, irrespective of the luteinizing hormone (LH) levels. This has led to a prevalence as high as 50% been quoted in some studies. However, the European Male Aging Study (EMAS) has demonstrated two distinct groups of older men with low total T (14). The majority of older men were found to have low T associated with low-normal luteinizing hormone. This is not independently associated with aging *per se* but is mediated indirectly via age-related non-gonadal co-morbidities, including obesity, and increased visceral adiposity. Only a small number of older men (2.1%) had low T with high LH, in keeping with primary testicular insufficiency. This specific primary hypogonadism profile has been directly associated with both aging and metrics of ill health.

On the other hand, it is imperative that the diagnostic evaluation of male hypogonadism be corroborated with signs and symptoms (25). However, there is substantial overlap between symptoms arising from chronic diseases and hypogonadism, posing significant challenge to determining clinically relevant LOH (14). Indeed, men reported hypogonadal symptoms frequently have T concentrations in the eugondal ranges (26). Moreover, the clinical significance of borderline or modestly low T levels typically seen in LOH is often hard to ascertain.

To address some of these gaps, EMAS investigators established a set of minimum criteria (14). In this study, 32 candidate symptoms were shortlisted, and after reductive analysis, only the co-occurrence of three sexual symptoms (decreased morning erection, poor libido, erectile dysfunction) and low T level (total T < 11 nmol/L and free T < 220 pmol/L) had consistent syndromic association. With that, the overall prevalence of LOH in EMAS population was determined to be 2.1%, widely believed to be the most accurate estimate hitherto, lower than previous studies using less stringent criteria (26). Stratifying by age groups, <1% of men aged <60 years, 3.2% of men aged 60–69 years, and 5.1% of men aged 70–79 years met the proposed criteria.

## The classification of LOH According to LH Level and Associated Risk Factors

The hypothalamus-pituitary- testicular (HPT) axis is tightly regulated in an interdependent fashion to maintain hormonal homeostasis. In hypogonadism, the gonadotropins can either be elevated (primary hypogonadism) or low/normal (secondary hypogonadism). In EMAS, subjects are classified into primary hypogonadism (LH > 9.4 u/L, T < 10.5 nmol/L), secondary hypogonadism (LH ≤ 9.4 u/L, T < 10.5 nmol/L) or compensated (primary) hypogonadism (LH > 9.4 u/L, T ≥ 10.5 nmol/L) (11). Through this approach, unique clinical characteristics and risk factors were identified in each subgroup.

Primary hypogonadism was found to be uncommon in the study. It affected only 2% of the entire cohort and had a low annual incidence of 0.2% (27). At-risk men had poorer baseline physical function, and suffered from deterioration in erectile function, vigor and hemoglobin as they progressed to hypogonadism. Advanced age (>70 years) and comorbidities were strongly associated with increased risk of primary hypogonadism, with an odds ratio of 12.5 and 4.24, respectively. The serum T concentrations continued to decline with time with little sign of recovery. For the minority whom T levels returned to eugonadal range, the mean LH levels remained persistently elevated to the same degree, indicating persistent Leydig cell failure.

Secondary hypogonadism accounted for majority (85.5%) of older men with low T, with an annual incidence of 1.6% (11). The mean LH level was not different from that of eugonadal men, indicating a failure in the compensatory hypothalamic-pituitary axis. Unlike primary hypogonadism, there was no significant relationship between the prevalence of secondary hypogonadism and aging. Instead, obesity emerged to be the most potent risk factor (14, 15), with a lesser contribution by comorbidities. Therefore, secondary hypogonadism represents a state of functional HPT suppression driven principally by obesity and poor health, rather than chronological aging.

The third classification was compensated hypogonadism, present in close to 10% of the study cohort. This group of men had normal circulating total T concentration and raised LH level. They exhibited some clinical features in keeping with primary hypogonadism (27), making it a clinically relevant entity. Despite being relatively common, progression to hypogonadism range of T concentration was very infrequent, suggesting that most men in this group could retain the capacity to sustain adequate T levels.

## Management of LOH

Subtyping LOH according to both T and LH levels provides useful clinical information in elucidating the underlying etiology, and allows management to be tailored accordingly. For LOH due to testicular failure (primary hypogoadism), T treatment could be used to improve anemia, sexual activity and libido in older men (28–34). However, T therapy was found to have no significant impact on energy level, physical function, weight, or cognitive function among older men with LOH (28, 35–40). Despite the reassuring data from majority of interventional trials with regards to short term safety (41–44), a meta-analysis of 27 placebo-controlled trials has concluded that T therapy was associated with an increased cardiovascular risk, with an odds ratio of 1.54 (95% confidence interval, 1.09 to 2.18) (45). Furthermore, T therapy is associated with increased hematocrit, serum concentrations of prostate-specific antigen (PSA) and prostate volume, as well as gynecomastia and secondary infertility. Hence, T therapy should only be considered after careful consideration of the risks and benefits, while bearing in mind that the cardiovascular safety profile of T therapy in this population has yet to be fully established. Ongoing surveillance of hematocrit and prostate specific antigen is also required whilst on T treatment (24).

On the other hand, human chorionic gonadotropin (HCG) may have a therapeutic role in LOH (46, 47). HCG therapy is known to increase serum testosterone concentration and preserve global activity of the testis (e.g., fertility and insulin-like factor 3 production) (48, 49). A clinical trial comparing 6-months HCG vs. T therapy in LOH has demonstrated higher 25-OH-vitamin D and lower serum estradiol concentrations in men treated with HCG (47). The prostate volume and hematocrit level were also significantly lower compared to the groups treated with T (47). The Leydig cells have been shown to contribute to the 25-hydroxylation of vitamin D and a higher 25-OH-vitamin D level may reflect improved Leydig cell function following HCG treatment (50). Hence, HCG therapy may have a favorable profile in LOH but larger safety and efficacy trials would be required to determine if HCG could be used as a long-term therapy in LOH.

It should be emphasized that obesity and co-morbidities underlies most cases of low T in older men with secondray hypogonadism, and thus, lifestyle intervention and cardiometabolic risk reduction should be the first line treatment for this cohort of patients. Notably, the potential for reversal to eugonadism in secondary hypogonadism is promising for obese men; nearly half of the men recovered their T levels over a period of ~4 years, predicted by attainment of healthier weight (51).

## Conclusion

Establishing the diagnosis of LOH remains a conundrum in clinical practice because of imprecise criteria and confounding factors relating to health alterations in old age. Nonetheless, if we define LOH as age-related primary testicular failure, only a minority of men appears to be affected. While studies have demonstrated some positive effects of T therapy, the clinical meaningfulness of these findings remains debatable. Moreover, the absence of long-term cardiovascular safety data continues to be an area of concern and controversy.

Hence, we suggest that future interventional trials for LOH should aim at older men with primary testicular failure, or classify the study cohorts according to LH levels so that a more clinically meaningful risk-benefit stratification can be elicited. This will clarify the safety and benefit profile of T therapy or other treatments in LOH and inform decision of the most appropriate management for LOH in men.

## Author Contributions

DS wrote the first draft of the paper. EG amended and rewrote the paper so that it matches the opinion style (paper was first submitted as review). EG produced the figure.

### Conflict of Interest Statement

The authors declare that the research was conducted in the absence of any commercial or financial relationships that could be construed as a potential conflict of interest.
